# They built this city—construction workers injured in Delhi, India: cross-sectional analysis of First Information Reports of the Delhi Police 2016–2018

**DOI:** 10.1186/s40621-022-00388-4

**Published:** 2022-07-21

**Authors:** Phil Edwards, Sajjan Yadav, Jonathan Bartlett, John Porter

**Affiliations:** 1 Department of Population Health, London School of Hygiene and Tropical Medicine, Keppel Street, London, WC1E 7HT UK; 2 Department of Expenditure, Ministry of Finance, Government of India, North Block, New Delhi, India; 3 Department of Mathematical Sciences, University of Bath, Claverton Down, Bath, BA2 7AY UK; 4 Department of Global Health and Development, London School of Hygiene and Tropical Medicine, Keppel Street, London, WC1E 7HT UK

**Keywords:** India, Construction, Injuries, Risk factors

## Abstract

**Background:**

Construction workers are 3–4 times more likely than other workers to die from accidents at work—however, in the developing world, the risks associated with construction work may be 6 times greater. India does not publish occupational injury statistics, and so little is known about construction workers injured. We aimed to use Indian police records to describe the epidemiology of construction site injuries in Delhi and to thus generate knowledge that may help to control the burden of injuries to construction workers in India and in other developing countries.

**Methods:**

This was a cross-sectional analysis of accident records maintained by the Delhi Police. We included all construction workers reported to have been killed or injured in construction site accidents in Delhi during the period 2016–2018. We used multivariable logistic regression models to investigate associations between injury severity (fatal vs. non-fatal injury) and exposure variables whilst adjusting for a priori risk factors. We also estimated the number of Delhi construction workers in total and by trade to generate estimates of worker injury rates per 100,000 workers per year.

**Results:**

There were 929 construction site accidents within the study period, in which 1,217 workers and children were reported to have sustained injuries: 356 (29%) were fatal and 861 (71%) were non-fatal. One-eighth of injuries were sustained by females. Most occurred in the Rainy season; most were sustained during the construction of buildings. The most frequent causes were the collapse of an old building, the collapse of a new building under construction, and electric shocks. Electricians were more likely than unskilled workers to suffer a fatal injury (adjOR 2.5; 95% CI: 0.87–6.97), and there were more electrical shocks than electricians injured. The odds of a fatal injury were statistically significantly lower in Central districts than in the less developed, peripheral districts.

**Conclusions:**

Construction site injuries are an unintended health impact of urbanisation. Women undertake manual work alongside men on construction sites in Delhi, and many suffer injuries as a consequence: an eighth of the injuries were sustained by females. Children accompanying their working parents on construction sites are also at risk. Two main hazards to construction workers in Delhi were building collapses and electrical shocks. Electricians were over twice as likely as unskilled workers to suffer a fatal injury, and electrical work would appear to be undertaken by a multitude of occupations. As the global urban population increases over the coming decades, so too will the burden of injuries to construction workers. The introduction and enforcement of occupational safety, health, and working conditions laws in India and in other rapidly developing countries will be necessary to help to control this injury burden to construction workers.

## Introduction

### Background

The urban population of the world is predicted to reach 5 billion in 2028 and 6 billion in 2041 (United Nations 2019). This urban growth will be due to rural–urban migration, geographic expansion of urban settlements, and the transformation of rural localities into urban settlements (United Nations 2019). These transformations will require construction on an unprecedented scale. One witness to such rapid transformation is India—its population has more than tripled since 1950 to 1.35 billion, and its urban population reached 34 per cent in 2018 (United Nations 2019). Delhi, its capital city, is one of the world’s fastest growing megacities (i.e. with over 10 million inhabitants) (Dhar Chakrabarti 2022). Here, the construction sector provides the main alternative to agricultural work—seasonal migration to and from construction is widespread in India and construction work remains the second-largest employer of women in the country behind agriculture (Bowers 2019); In Delhi, *‘it is the poor and illiterate labour force from the villages which has kept the city growing, and which keeps the city going.’* (Dhar Chakrabarti 2022).

Construction work can be hazardous in most settings—construction workers are 3–4 times more likely than other workers to die from accidents at work—however, in the developing world, the risks associated with construction work may be 3–6 times greater (International Labour Organisation 2022a).

We conducted a brief review of the literature to identify risk factors for construction site injuries: age has been found to be associated with risk of construction site injury in the developed and developing world (Calkins et al. 2019; Schoenfisch et al. 2010; Reese and Eidson 2006; Mučenski et al. 2015; Dong et al. 2010; Amissah et al. 2019; Kiconco et al. 2017; Kalam 2017; Chau et al. 2004; Camino López et al. 2018; López et al. 2008; Schwatka et al. 2012; Jackson and Loomis 2002); migrant workers are often disadvantaged in the labour market, due to language barriers, cultural differences, and lower average levels of education, and these may manifest in increased injury risk (Roelofs et al. 2011; Arditi et al. 2003; Amick et al. 2015; International Labour Organisation 2022b); trade specialisation has been found to be associated with the risk of injury among frontline building construction workers (Amissah et al. 2019); electrocution is one of the three leading causes of death for construction workers in the developed world (Kisner and Fosbroke 1994); and occupational accidents may be associated with a ‘construction season’ (Szóstak 2019).

As India does not report or publish statistics on occupational injuries (Hämäläinen et al. 2006), relatively little is known about construction workers injured in Delhi. We have previously shown that Indian Police records may be used as the basis of an injury surveillance system (Yadav et al. 2020a), and we have used these data to estimate annual construction site injury rates per 100,000 workers in Delhi (146.5 (95%CI 137.7–155.6) in males and 82.26 (95%CI 57.92–113.39) in females) (Yadav et al. 2021).

In this study, we aimed to use these police records to describe the epidemiology of construction site injuries in Delhi and to assess risk factors for fatal injuries to learn more about this direct health impact of urbanisation on those who build the cities, and to thus generate knowledge that may help to control the burden of injuries to construction workers in India and in other developing countries. Specifically, this current study adds the descriptive epidemiology of construction site injuries in terms of person, place, and time to the previous studies (Yadav et al. 2020a, 2021).

## Methods

### Aims

Our specific aims were to:

- Describe the epidemiology of construction site injuries in Delhi;
- Assess risk factors for fatal construction site injuries in Delhi;

We hypothesised that:

H1: Electricians are at 3 times greater risk of injury than ‘unskilled’ workers due to the hazards of working with electricity (Kisner and Fosbroke 1994);

H2: The odds of a fatal injury are higher in migrant than native workers (due to language barriers, cultural differences, lower average levels of education, a higher proportion of exposure of unskilled workers to new technology, high-risk jobs, and stress) (Roelofs et al. 2011; Arditi et al. 2003; Amick et al. 2015; International Labour Organisation 2022b).

### Study design

This was a cross-sectional analysis of data extracted from the First Information Reports (FIRs) of the Delhi Police (Yadav et al. 2020a). We also estimated the number of Delhi construction workers in total and by trade to generate estimates of worker injury rates.

### Setting

This study was conducted in Delhi, the capital of India, and it included all construction workers reported to have been killed or injured in construction site accidents during a three-year period from 1 January 2016 to 31 December 2018.

### Participants

All persons who were reported to the Delhi police as killed or injured in an accident at a building or other construction site during the study period were included (The Building and other Construction Workers (Regulation of Employment and Conditions of Service) 1996).

### Variables

#### Outcome

*Injury severity—* injuries were categorised as ‘fatal’ or ‘non-fatal’.

The injuries sustained were documented in the police records, but we chose to focus on outcomes of Public Health importance (i.e. vital status) rather than to investigate fractures and tissue damage, etc.

#### Exposures

We investigated a priori risk factors for construction site injuries in Delhi*,* identified in our brief review of the literature:

(i) *Age*
(ii) *Migrant workers*
(iii) *Trade*
(iv) *Season*

We investigated these risk factors using the following categorisations:

*Age group*—For analysis of injury risk by age, we used the age categories advised in the World Health Organization (WHO) Injury Surveillance Guidelines: < 5 years; 5–14 years; 15–19 years; 20–21 years; 22–44 years; 45–64 years; and > 64 years (World Health Organization 2001).

*Residence*—For analysis of injury risk by place of permanent residence of the injured person, we used two categories of residence: (i) native of Delhi and (ii) migrant from other state of India or from another country.

*Trade*—For analysis of injury risk by trade, we used the categories: Unskilled worker; Mason; Carpenter; Plumber; Electrician; and Other (e.g. painter).

*Season*—to investigate seasonal variations in construction injuries, we analysed the numbers of injuries sustained in three seasons: Summer (March to June), Rainy (July to October), and Winter (November to February).

**Fig. 1:**
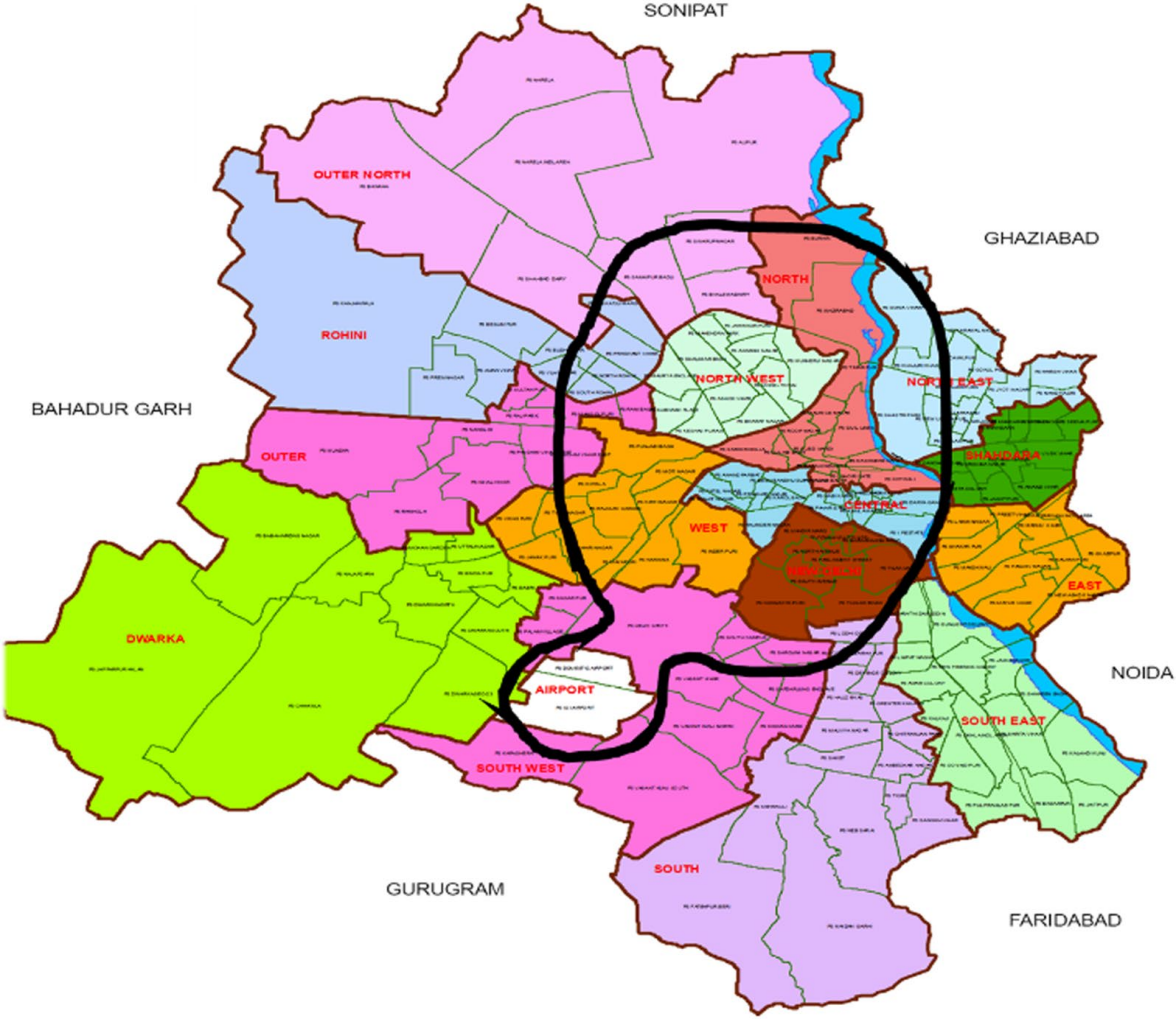
Map of Delhi (Central districts shown by black line)

*Geography*—For analysis of injury risk by geographical location of the accident, we used two geographical groupings (see Fig. 1) of the districts of Delhi: **Central** districts (New Delhi, Central, West, North, North West, and IGI Airport) and **Peripheral** districts (Outer, Rohini, Shahdara, South, South East, South West, Dwarka, East, Metro, and North East).

### Data sources

In India, information relating to an accident, whether received orally or in writing, must be recorded in a book by the officer in-charge of a police station, in a prescribed format, commonly known as the First Information Report (FIR) (Code 2019). We have previously shown that these FIRs may be used as the basis of an injury surveillance system (Yadav et al. 2020a); in this study, we used these data for an epidemiological investigation into construction site injuries in Delhi.

### Data extraction

We first obtained data from the Delhi police that identified all accidents reported to them, namely FIR number, year, name of police station, and name of district. We downloaded the FIR documents for each accident from the Delhi Police website (Delhi Police Shanti Sewa Nyaya 2022). We screened these FIR documents to identify all accidents reported at building and other construction sites. If an FIR document was unavailable on the Delhi Police website for any accident, we obtained the document directly from the police station concerned. We then extracted data from the FIRs into an MS-Excel worksheet; data recorded on the FIRs were narratives that we reviewed and categorised using a data extraction tool (Yadav et al. 2020a). The police determined the causes of accidents based on the information gathered from the statements of victims and witnesses of accidents. In the case of fatal accidents, data were provided by witnesses of the accident; these included co-workers, supervisors, managerial personnel and neighbours. First responders like fire and police personnel also added to this information.

### Denominators

We have previously estimated the size of the construction workforce in Delhi in 2017 as 756,938 workers (711,960 males and 44,978 females) (Yadav and P, 2021). In this study, we estimated the size of the construction workforce in Delhi in 2017 by trade, by applying estimates of the proportions of workers in the Construction Sector in India in 2022 by trade (Hajela 2012).

### Statistical methods

We examined characteristics of construction workers killed or injured in construction site accidents stratified by sex.

Where denominators were available, we estimated injury rates per 100,000 workers per year with 95% confidence intervals assuming Poisson-distributed counts of injuries (i.e. using the *cii means, Poisson* command in Stata). We estimated the annual numerator by dividing the total number of injuries reported during the three-year period by 3. We excluded all children (i.e. persons aged under 15 years) from the numerators when estimating rates.

We used multivariable logistic regression models to investigate any associations between injury severity (fatal vs. non-fatal injury) and each exposure variable whilst adjusting for all a priori risk factors. These associations were thus quantified by odds ratios which indicate whether an injury was more or less likely to be fatal with the exposure under investigation.

Some variables had missing values. Logistic regression models were fitted using a complete case analysis. The odds ratio for a covariate estimated in this way is an unbiased estimate unless the probability of having complete data depends on both the covariate in question and the outcome, in which case bias can arise (Bartlett et al. 2015): we fitted additional logistic regression models with a binary indicator of whether an individual had complete data as the outcome to investigate these assumptions. All analyses were conducted using Stata 16.1 (StataCorp 2019).

### Ethics approval and consent to participate

This study was approved by the London School of Hygiene and Tropical Medicine (LSHTM) Observational Research Ethics Committee (see LSHTM Ethics Reference number 15992, dated 26 November 2018). All methods were performed during the study in accordance with the relevant guidelines and regulations of LSHTM. The data used in this study were those provided by the injured persons to the Delhi Police. The study was also approved by the Ethics Committee of Dr. Baba Saheb Ambedkar Medical College and Hospital, Delhi. As all data were provided in an anonymised format and permission to use the data was granted by the Delhi Police, the Committee waived off the need to obtain informed consent of individuals whose anonymised data were included in the study.

## Results

### Construction site accidents and workers injured

**Fig. 2:**
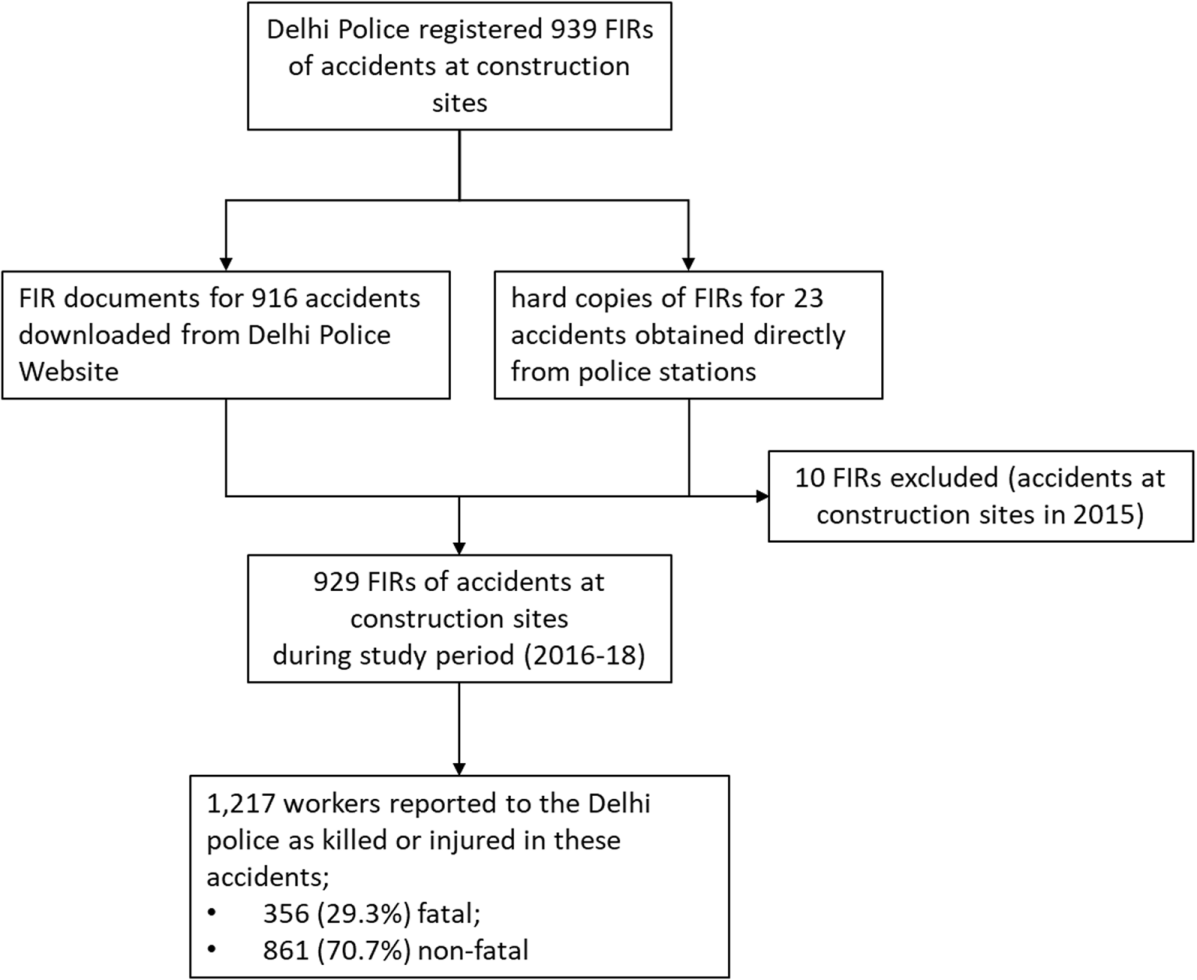
Study flow

During the study period, Delhi Police registered 939 FIRs of accidents at construction sites. The FIR documents were available from the Delhi Police website for 916 accidents; hard copies of FIR documents were obtained directly from the police station at which they were registered for 23 accidents. Ten FIRs were excluded because they were reports of accidents that had occurred outside of the study period. In the 929 incidents within the study period, a total 1,217 people were reported to have sustained injuries: 356 (29.3%) were fatal, and 861 (70.7%) were non-fatal (Fig. 2).

### Denominators

Our estimates of the size of the construction workforce in Delhi in 2017 by trade are shown in Table 1.

**Table 1:** Estimated size of the construction workforce in Delhi in 2017 by trade

Trade	Workers
Carpenter	32,950
Electrician	8,237
Mason	24,712
Plumber	20,602
Unskilled worker	662,443
Other (e.g. painters)	7,994
Total	756,938

### Descriptive results

#### Year

The numbers of accidents and workers injured declined over the study period (Table 2).

**Table 2:** Construction site accidents and workers injured in Delhi, 2016–2018

Year	Accidents	Workers injured		
		Non-fatal	Fatal	Total
2016	357	390	138	528
2017	318	263	109	372
2018	254	208	109	317
Total	929	861	356	1217

Table 3 shows the characteristics of construction workers injured in Delhi, 2016–2018.

**Table 3:** Characteristics of construction workers injured in Delhi, 2016–2018

	Male		Female			Total	*%*	*Injury rate*
	Non-fatal	Fatal	Non-fatal		Fatal			*Per year per 100,000 (95% CI)*
Age								
< 5	13	5	9		4	31	*3*	
5–14	29	11	18		3	61	*5*	
15–19	44	15	10			69	*6*	
20–21	44	24	3			71	*6*	
22–44	341	154	33		10	538	*44*	
45–64	85	37	10		4	136	*11*	
> 64	9	3	4			16	*1*	
*Age missing*	164	83	45		3	295	*24*	
Total	729	332	132		24	1217		
Total (adults)	687	316	105		17	1125		*49.5 (44.7–54.8)*
Season								
Summer (March–June)	285	125	40	7		457	*38*	
Rainy (July–October)	282	129	61	9		481	*40*	
Winter (November–February)	162	78	31	8		279	*22*	
Residence								
Native to Delhi	432	158	109	15		714	*59*	
Migrant	229	127	21	8		385	*32*	
*Residence missing*	68	47	2	1		118	*9*	
Trade								
Carpenter	6	2				8	*1*	*9.1 (1.9–26.6)*
Electrician	8	9				17	*1*	*72.8 (26.7–158.6)*
Mason	63	33				96	*8*	*129.5 (88.6–182.8)*
Plumber	11	3				14	*1*	*22.7 (7.9–56.6)*
Unskilled worker	401	207	12	6		626	*51*	*31.5 (27.4–36.1)*
Other (e.g. painters)	117	53	64	6		240	*20*	*1,000.8 (793.5–1,245.5)*
*Trade Missing*	123	25	56	12		216	*18*	*–*
Construction type								
Building	620	276	120	22		1038	*85*	
Erection of a temporary structure (tent/dome)	16	7				23	*2*	
Flyover/bridge/underpass	7	6				13	*1*	
Metro rail works	15	11	2			28	*2*	
Power generation and distribution works	3	4	1			8	*1*	
Road/street	1	1				2	*0.2*	
Sewerage works	7	3	1			11	*1*	
Telecom and television works	3	2				5	*0.4*	
Water supply related works	1	5				6	*0.5*	
Others	28	6	5			39	*3*	
*Missing*	28	11	3	2		44	*4*	
Geography								
Central districts	262	97	41	9		409	*343*	
Peripheral districts	467	235	91	15		808	*66*	
Cause								
Accidental fall in water		4				4	*0.3*	
Accidental fall of bricks/building material	30	9	10	2		51	*4*	
Accidental fall of other object/equipment	30	14	11			55	*5*	
Accidental hit by vehicle or moving machine	11	4				15	*1*	
Accidental injury by stationery machine/equipment	3	3				6	*0.5*	
Break of rope/harness	10	5				15	*1*	
Collapse of old building	165	41	52	10		268	*22*	
Collapse of roof/wall/part of under-construction building/building material	153	19	53	4		229	*19*	
Collapse of scaffolding/platform	54	10	2			66	*5*	
Collapse of surrounding earth of a pit/basement/tunnel	8	7		1		16	*1*	
Electrical shock	59	68				127	*10*	
Fire	2					2	*0.2*	
Gap in building/stairs	1	1				2	*0.2*	
Lack of barricade/railing/cover	31	36	3	5		75	*6*	
Lifting of heavy object		1				1	*0.1*	
Slipping of ladder	8	9				17	*1*	
Slipping of person	54	37		1		92	*8*	
Other	5	1				6	*0.5*	
Missing	105	63	1	1		170	*14*	
Total	729	332	132	24		1217		

#### Sex

One-eighth of the injuries were sustained by females.

#### Age

Information on age was missing in 295 (24%) cases. The greatest number of injuries was in workers aged 22–44 years and lowest in workers aged over 64 years. Of the 1,217 people reported to have sustained injuries, 92 (8%) were children.

#### Season

The number of injuries was highest in the Rainy season and lowest in the Winter.

#### Residence

Information on residence was missing in 118 (9%) cases. Approximately two-thirds (65%) of injured workers were residents of Delhi and approximately one-third (35%) were migrants.

#### Trade

Information on trade was missing in 216 (18%) cases. There were more injuries among unskilled workers than among carpenters, electricians, and plumbers. Seventeen electricians were injured.

#### Construction type

Information on the type of construction was missing in 44 (4%) cases. Most injuries were sustained during the construction of buildings, as opposed to during works on roads, rail, or utilities.

#### Geography

Two-thirds of the injuries occurred in the Peripheral districts of Delhi and one-third occurred in the Central districts of Delhi. The geographical distribution was similar for fatal and non-fatal injuries, although the prevalence of fatal injuries was marginally higher in the peripheral districts (70%).

#### Causes

Information on the cause of the accident was missing in 170 (14%) cases. The most frequent causes of accidents were: the collapse of an old building (268 (22%) cases); the collapse of the roof, wall, or other part of a new building under construction (229 (19%) cases); and electrical shocks (127 (10%) cases (Table 3).

#### Injury rates

We estimate that the annual injury rate was 49.5 (95% CI 44.7–54.8) per 100,000 workers. The annual injury rate was highest among workers whose trade was recorded as ‘Other’ (e.g. painters), with 1,000.8 (95%CI 793.5–1,245.5) per 100,000 workers, and it was lowest among Carpenters with 9.1 (95% CI 1.9–26.6) per 100,000 workers.

### Multivariable results

There was no evidence that the probability of being a complete case was associated with the outcome (OR 1.03 fatal vs. non-fatal; 95% CI 0.80–1.33, p = 0.81); missingness being independent of outcome means that the odds ratios estimated by our complete case multivariable logistic regression are unbiased (Bartlett et al. 2015). Missingness was also unrelated to any of the fully observed or almost fully observed variables which implies that missingness was random.

Table 4 shows the results of our multivariable analysis of risk factors for a fatal construction site injury. Females were half as likely as males to suffer a fatal injury (adjOR 0.5; 95% CI 0.24–1.05). Electricians were more likely than unskilled workers to suffer a fatal injury (adjOR 2.5; 95% CI 0.87–6.97). Compared with the Peripheral districts of Delhi, the odds of a fatal injury were statistically significantly lower in the Central districts. No season was more likely to present a risk for fatal injuries than any other.

**Table 4:** Risk factors for fatal construction site injuries in Delhi

	N^§^	Odds ratio^¥^	95% confidence interval	P-value
Sex				
Male	659	Referent	–	–
Female	66	0.50	0.24–1.05	0.067
Age group				
< 5	21	1.75	0.60–5.08	0.31
5–14	51	1.3	0.60–2.79	0.504
15–19	52	0.66	0.32–1.33	0.243
20–21	62	1.27	0.71–2.25	0.417
22–44	441	Referent	–	–
45–64	92	.94	0.56–1.56	0.806
> 64	6	1.33	0.24–7.52	0.747
Residence				
Native to Delhi	431	Referent	–	–
Migrant	294	1.2	0.86–1.67	0.282
Season				
Summer (March–June)	270	Referent	–	–
Rainy (July–October)	267	0.92	0.63–1.35	0.683
Winter (November–February)	188	1.1	0.73–1.66	0.658
Trade				
Unskilled	449	Referent	–	–
Electrician	15	2.5	0.87–6.97	0.091
Other (e.g. painters)	261	0.64	0.43–0.95	0.029
Geography				
Peripheral district	482	Referent	–	–
Central district	243	0.69	0.48–0.98	0.039

^§^Number of observations included in the complete case analysis

^¥^OR adjusted for all other exposures in the table

## Discussion

### Principal findings

The two main hazards to construction workers in Delhi were building collapses and electrical shocks. Electricians were over twice as likely as unskilled workers to suffer a fatal injury. One-eighth of the injuries were sustained by females. Females were half as likely as males to suffer a fatal injury.

### Strengths and weaknesses of the study

This is the first study of the epidemiology of construction site injuries in Delhi. A further strength of this study is that it covered the entire city of Delhi and it includes injuries reported over a three-year period—it has therefore established a baseline against which future construction site injury reports may be compared. Another strength is that our study was sufficiently powered to show differences in injury risk—for our sample size calculation, we first estimated the injury rate of unskilled construction site workers in India by using the non-fatal occupational injury rate in neighbouring Pakistan in 2018 (1,136 non-fatal injuries per 100,000 workers) (International Labour Organisation 2022c). We had hypothesised that electricians are at 3 times greater risk of injury than unskilled workers due to the hazards of working with electricity (Kisner and Fosbroke 1994); thus, we hypothesised that the non-fatal injury rates are 1,136 per 100,000 unskilled workers versus 3,408 non-fatal injuries per 100,000 electricians (i.e. 0.01136 versus 0.003408, respectively). As there were an estimated 662,443 unskilled workers and 8,237 electricians in Delhi in 2017 (Table 1), our study had over 95% power to detect this hypothesised difference in non-fatal injury rates at the 1% level of significance.

A major limitation of this study is the under-reporting of construction site injuries to the police (approximately two‑thirds of injuries are not reported to the police) (Yadav et al. 2020b). This may mean that our study under-represents the more precariously employed of workers (Kreshpaj et al. 2022); we may, therefore, have under-estimated the relative risks to some groups of workers, such as migrant workers. A further limitation is that information on age, residence, and trade was missing in over 5% of cases. However, complete case analysis multivariate logistic regression gives unbiased odds ratio estimates if missingness (i.e. no value provided for the variable within the dataset of the incident events) is unrelated to the outcome variable (fatal vs. non-fatal injury) (Bartlett et al. 2015), and we found no evidence that it was related.

Another limitation of our study is that we used estimates of the size of the construction workforce in Delhi in 2017 by trade, in order to estimate injury rates per 100,000 workers per year by trade. Our estimate of the annual injury rate among workers whose trade was recorded as ‘Other’ (e.g. painters) was approximately 20 times higher than the average injury rate, which is not plausible and most likely due to a mismatch between numerator and denominator in this case: our estimates of the size of the construction workforce in Delhi in 2017 by trade are therefore probably inaccurate, and our estimates of injury rates per 100,000 workers per year by trade are therefore also inaccurate. Furthermore, our estimates of the injury rates of unskilled workers and electricians in Delhi were far lower than those we had expected and hypothesised, based on rates in neighbouring Pakistan. These differences are also most likely due to a mismatch between numerators and denominators, due to under-reporting of injuries and inaccuracies in our estimated denominators by trade.

Under-reporting of accidents is a common limitation in many injury surveillance systems and must be borne in mind by any public health or injury prevention practitioner wishing to use the results: they may be used for the planning of preventive interventions—i.e. they are indicative of *where* and *to whom* fatal injuries will occur, but they should not be relied upon for the evaluation of preventive interventions, because changes after a preventive intervention may be due to changes in the reporting of injuries to the police, rather than due to the success or failure of an intervention.

### Strengths and weaknesses in relation to other studies

As we have already indicated, relatively little was previously known about construction workers injured in Delhi. We could only identify one other study—a retrospective study of 145 construction site accident autopsies in South Delhi during the period 1996–2002 (Rautji et al. 2005). As in our study, the majority of fatal injuries were male (in our study 332 (93.3%) of the 356 fatal injuries were male, similar to the 93.8% found in the autopsy study); most injured workers were aged around 22–44 years, and few were aged over 64 years; around one-fifth of injuries were caused by the collapse of an old building, or the collapse of the roof, wall, or other part of a new building under construction. (In our study, 92 (26%) of the 356 fatal injuries were caused by a collapse, similar to the 20% of workers being trapped inside falling buildings or masonry found in the autopsy study.)

One notable difference, however, was that we found a higher proportion of electrocutions than did the study of autopsies. (In our study, 68 (19%) of the 356 fatal injuries were electrocutions, compared to the 3.45% found in the autopsy study.) However, as electrocution is one of the three leading causes of death for construction workers in the developed world (Kisner and Fosbroke 1994), we consider that our estimate of 19% of fatalities are due to  electrocution is likely to be more accurate than the 3.45% found in the autopsy study.

A study in Ethiopia also attributed the burden of injuries there to its rapid urban development (Ali et al. 2020).

Our estimate of the annual injury rate (49.5 per 100,000 workers) in Delhi is comparable with the rates reported in Ukraine (54 per 100,000 workers), Belarus (51 per 100,000 workers), and Armenia (50 per 100,000 workers), but is less than one-tenth of the rates reported in many highly developed countries (e.g. UK—760 per 100,000 workers, the USA—900 per 100,000 workers, and Germany—1,811 per 100,000 workers) (International Labour Organisation 2022c). These differences are likely to be due to the under-reporting of construction site injuries to the police in Delhi, as we have discussed above.

### Meaning of the study: possible mechanisms and implications for clinicians or policymakers

As with other studies in injury epidemiology, we suggest that our study results likely reflect differences between individuals in amounts of exposure to hazards—i.e. most construction workers in Delhi are likely to be unskilled males aged 22–44 years, native to Delhi, working on the construction of buildings in the Peripheral districts of Delhi. However, as the global urban population rapidly increases over the coming decades, so too will the burden of injuries to construction workers in the developing countries. Our study contributes knowledge that may help to control this burden—the 2 main hazards to construction workers in developing countries are likely to be building collapses and electrocution.

Building collapses could be reduced if competent professionals (i.e. surveyors and structural engineers) are employed to: identify any faults that may be present in existing structures and to advise on the sequencing of demolition, and on any temporary works that are necessary (e.g. propping of load-bearing walls). Electrocutions could be reduced if measures are put in place to ensure that the works are properly planned by competent managers and only undertaken by competent electricians (that 17 electricians were injured, but ‘electrical shock’ was the cause of 127 injuries would suggest that electrical work is undertaken by a multitude of occupations). Furthermore, safe systems of work should be implemented to eliminate or reduce the electrical hazard (e.g. cutting off electrical supply, locking off circuits that are being worked on, and restricting access to areas where circuits are live).

Nearly 50 years ago in the UK, the Health and Safety at Work Act was introduced that placed a requirement on employers to remove or reduce risk ‘as far as is reasonably practical’ (Robertson 2015). In the 40 years since it was introduced, work fatalities reduced by 85%, and non-fatal injuries reduced by 77% (Robertson 2015). In India, the Occupational Safety, Health and Working Conditions Code introduced in 2020 appears to place a similar requirement on employers (Safety 2022). With sufficient funding and support for its enforcement, we might expect to see similar reductions in construction site injuries as those seen in the UK. As in the UK, building contractors in India and in other developing countries should be expected to apply a hierarchy of control measures to reduce the risks posed by the hazards present (HSE 2022): (i) Eliminate—physically remove the hazard; (ii) Reduce—substitute the hazard; (iii) Isolate—segregate the hazard; (iv) Control—change how the workers perform their duties; (v) PPE—provide appropriate protective equipment (e.g. hard hats, safety boots, gloves, goggles) to help to reduce injury severity in the event of an accident; and (vi) Discipline—educate workers on appropriate control measures and reprimand workers if these are not correctly followed.

We found the odds of a fatal injury were statistically significantly higher in the Peripheral districts of Delhi than in the Central districts. Being peripheral and less populated, these districts may accommodate Delhi’s expansion through unplanned development and unauthorised, riskier constructions that deserve greater scrutiny by the local government officials responsible for enforcing the Occupational Safety, Health and Working Conditions Code in these districts.

### Unanswered questions and future research

As a major limitation of this study is due to the under-reporting of construction site injuries to the police, further research is needed to identify the reasons that some injuries are not reported. This could lead to development of a strategy to improve the completeness of reporting of construction site injuries in the future.

Qualitative research with migrant workers might usefully illuminate reasons why migrants may be more likely than native workers to suffer a fatal injury (i.e. the extent to which this is due to language barriers, cultural differences, lower average levels of education, exposure of unskilled workers to new technology, or being required to do the more hazardous jobs).

We found that some of the people reported to have sustained injuries were children (i.e. persons aged under 15 years). Some of these people may indeed be young workers, but further research could establish whether those people under 5 years are in fact children accompanying their parents. If they are young children, legislation in India already provides for the setting up of childcare facilities at construction sites where more than 50 female workers are ordinarily employed, but this legislation may need to be amended to make provision of childcare facilities mandatory at construction sites where *any* mothers are employed (Yadav et al. 2021).

This study demonstrates that Indian Police records may be used as the basis of an injury surveillance system. Further research into the strengths and weaknesses of this data source in injury surveillance is needed.

## Conclusions

This is the first study of the epidemiology of construction site injuries in Delhi. It is evident that women undertake manual work alongside men on construction sites in Delhi, and many suffer injuries as a consequence: one-eighth of the injuries were sustained by females. The two main hazards to construction workers in Delhi were building collapses and electrical shocks. Electricians were over twice as likely as unskilled workers to suffer a fatal injury. As the global urban population increases over the coming decades, so too will the burden of injuries to construction workers. The introduction and enforcement of occupational safety, health, and working conditions laws in India and in other rapidly developing countries will be necessary to help to control this injury burden to construction workers. Whether the ‘David’ of Occupational Safety Legislation will be a strong-enough match for the ‘Goliath’ of the Population Increase ‘Tsunami’ will critically depend on David getting sufficient buy-in from industry and its largely unskilled workforce.

## Data Availability

The datasets used and/or analysed during the current study are available from the corresponding author on reasonable request.

## Abbreviations

FIR: First Information Report
WHO: World Health Organization
LSHTM: London School of Hygiene and Tropical Medicine
PPE: Personal Protective Equipment
